# The Interaction Analysis between the Sympathetic and Parasympathetic Systems in CHF by Using Transfer Entropy Method

**DOI:** 10.3390/e20100795

**Published:** 2018-10-16

**Authors:** Daiyi Luo, Weifeng Pan, Yifan Li, Kaicheng Feng, Guanzheng Liu

**Affiliations:** 1 School of Biomedical Engineering, Sun Yat-sen University, Guangzhou 510275, China; 2 Key Laboratory of Sensing Technology and Biomedical Instrument of Guangdong Province, School of Engineering, Sun Yat-sen University, Guangzhou 510275, China; 3 Guangdong Provincial Engineering and Technology Centre of Advanced and Portable Medical Device, Guangzhou 510275, China

**Keywords:** congestive heart failure (CHF), heart rate variability (HRV), autonomic nervous system (ANS), interaction, sympathetic, parasympathetic, transfer entropy (TE)

## Abstract

Congestive heart failure (CHF) is a cardiovascular disease associated with autonomic dysfunction, where sympathovagal imbalance was reported in many studies using heart rate variability (HRV). To learn more about the dynamic interaction in the autonomic nervous system (ANS), we explored the directed interaction between the sympathetic nervous system (SNS) and the parasympathetic nervous system (PNS) with the help of transfer entropy (TE). This article included 24-h RR interval signals of 54 healthy subjects (31 males and 23 females, 61.38 ± 11.63 years old) and 44 CHF subjects (8 males and 2 females, 19 subjects’ gender were unknown, 55.51 ± 11.44 years old, 4 in class I, 8 in class II and 32 in class III~IV, according to the New York Heart Association Function Classification), obtained from the PhysioNet database and then segmented into 5-min non-overlapping epochs using cubic spline interpolation. For each segment in the normal group and CHF group, frequency-domain features included low-frequency (LF) power, high-frequency (HF) power and LF/HF ratio were extracted as classical estimators of autonomic activity. In the nonlinear domain, TE between LF and HF were calculated to quantify the information exchanging between SNS and PNS. Compared with the normal group, an extreme decrease in LF/HF ratio (*p* = 0.000) and extreme increases in both TE(LF→HF) (*p* = 0.000) and TE(HF→LF) (*p* = 0.000) in the CHF group were observed. Moreover, both in normal and CHF groups, TE(LF→HF) was a lot greater than TE(HF→LF) (*p* = 0.000), revealing that TE was able to distinguish the difference in the amount of directed information transfer among ANS. Extracted features were further applied in discriminating CHF using IBM SPSS Statistics discriminant analysis. The combination of the LF/HF ratio, TE(LF→HF) and TE(HF→LF) reached the highest screening accuracy (83.7%). Our results suggested that TE could serve as a complement to traditional index LF/HF in CHF screening.

## 1. Introduction

Congestive Heart Failure (CHF) refers to a chronic progressive condition, associated with the failure of the heart to pump blood in adequate volume to meet the requirements of the body [1]. CHF is related with autonomic nervous system (ANS) activities dysfunction [2], since neurohumoral modulation plays an important part in regulating cardiovascular function. To assess the autonomic cardiovascular control, heart rate variability (HRV) is commonly considered as a reliable noninvasive means to investigate autonomic modulation in the heart [3].

HRV features have been extensively studied as markers of risk factors of CHF. Traditionally, methods used to analyze HRV are focused on time and frequency domain analyses. Nolan et al. [2] identified a reduction in standard deviation of the RR intervals (SDNN) as an indicator of high-risk subgroups among CHF patients. Rao et al. [4] showed reduced standard deviation of the average RR interval (SDANN) in CHF patients compared with normal subjects. Ponikowski et al. [5] found HRV indices SDNN, SDANN, the mean of all 5-min standard deviations (SD) of RR intervals and low-frequency (LF) power capable to independently predict death in chronic congestive heart failure. Piccirillo et al. [6] also reported significantly lowered HRV measures in CHF patients and thus proposed a decrease of the LF power spectral component as a risk factor for sudden death. 

However, people come to realize that CHF is caused by multiple factors and thus HRV contains much more complex information about the nonlinear fluctuations. Therefore, more and more researches about CHF have focused on nonlinear analysis in chaotic, fractal and entropic manners. Poon and Merrill [7] confirmed a decrease of cardiac chaos and a higher degree of random variability in CHF. Makikallio et al. [8] studied the prognostic power of spectral and fractal analytic methods of HRV in CHF patients and found the reduced short-term fractal exponent a strong independent predictor of mortality. Liu et al. [9] presented an accurate approach to detect CHF based on the support vector machine and three nonstandard HRV measures, among which approximate entropy and sample entropy are used to compute the nonlinear measures.

Among CHF patients, about half have a dilated heart with systolic dysfunction, while the other half have a hypertrophied heart with diastolic dysfunction [10]. There are many studies focused on the changes of sympathetic and parasympathetic activity of ANS while little research has focused on estimating the directed information flows between the sympathetic nervous system (SNS) and the parasympathetic nervous system (PNS). The autonomic nervous system (ANS) modulates either systolic function or diastolic function. The normal modulation of ANS depends on the balance of excitation and inhibition between these two subsystems. Since an imbalance of ANS activity exists in CHF, flows of asymmetry-directed information among these two elements may illustrate the asymmetric interaction between the SNS and PNS. To understand the pathophysiology of CHF, assessment of ANS imbalance by analyzing HRV may be a useful approach [11,12]. 

As a nonlinear approach to analyze HRV, transfer entropy (TE) is capable of both distinguishing driving and responding elements and detecting asymmetry in the interaction among subsystems, with a solid foundation in information theory [13]. TE is an ideal tool to estimate the amount of information exchanging between the SNS and PNS [14], as SNS and PNS are not just simply playing an “opposite” role but also have complex dynamic interaction [15]. Moreover, considering that TE requires little previous assumption and ignores static correlations due to the common history, it is suitable to analyze ANS activity whose interacting mechanism is not yet comprehensively understood. Faes et al. [16] applied TE to evaluate nonlinear causality in cardio-vascular and cardio-respiratory control systems and suggested TE as a non-invasive tool for monitoring cardio-vascular and cardio-respiratory regulation. Marzbanrad et al. [17] revealed TE with delays as a novel tool to provide information on the fetal-maternal heart rate coupling strength and latency throughout gestation. More and more analysis of information transfer in neurophysiology and cardiovascular physiology [16,17,18,19] revealed TE as a powerful tool. Zheng et al. used TE among the low-frequency (LF) component and high-frequency (HF) components to study the interaction between SNS and PNS in sleep apnea patients [20].

Previous studies have shown that the LF component of RR intervals was able to reflect SNS activity [21] while HF component was able to reflect PNS activity [22,23]. Binkley et al. [24] used the power of LF and the power of HF to demonstrate the sympathetic and parasympathetic tone in CHF. In this paper, we used the concept that the LF component and HF component reflect the SNS and PNS activity, respectively. Besides using the LF/HF ratio to estimate the imbalance of ANS activity in CHF, we attempted to explore whether asymmetric information flows exist between SNS and PNS by employing TE to measure the directed dynamical interaction between LF and HF.

## 2. Materials and Methods 

**Figure 1:**
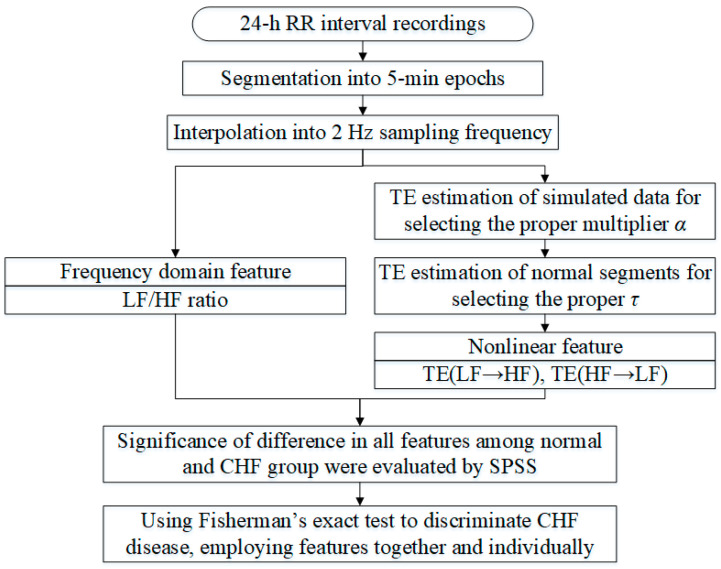
The flowchart of the signal processing of our study in this paper.

Figure 1 presents the flowchart of signal processing.

### 2.1. Data

The 24-h RR interval signals were both obtained from PhysioNet, consisting of data from 54 healthy subjects (31 males and 23 females, 61.38 ± 11.63 years old) and 44 CHF subjects (8 males and 2 females, 19 subjects’ genders were unknown, 55.51 ± 11.44 years old). All the recordings of normal people came from the Normal Sinus Rhythm RR Interval database (sampled in 128 Hz), while among the recordings of CHF subjects, 15 came from the Beth Israel Deaconess Medical Center (BIDMC) Congestive Heart Failure database (sampled in 250 Hz; this group of subjects was part of a larger study group with severe congestive heart failure despite treatment with digitalis glycosides, diuretics and one or more oral vasodilators [25]) and 29 from the Congestive Heart Failure (CHF) RR Interval database (sampled in 128 Hz; 10 subjects within this group were receiving constant doses of digoxin, diuretics, and angiotensin-converting enzyme inhibitors for at least 2 weeks before the signal recording. The cause of heart failure was idiopathic dilated cardiomyopathy in six patients and ischemic cardiomyopathy in four patients. Information about the remaining 19 subjects was unknown). According to the New York Heart Association (NYHA) Functional Classification, the 44 CHF subjects included 4 in class I, 8 in class II and 32 in class III~IV. Table 1 shows more details about the data used. 

For every 24-h recording, the first and the RR interval were abandoned and any RR interval longer than 3 s was also deleted for signal denoising. Then, the remaining data was divided into multiple non-overlapping 5-min segments in order to apply standard short-term HRV analysis. The sampling frequency of the 5-min segments was inverted to 2 Hz via cubic spline interpolation [26].

### 2.2. Feature Extraction

#### 2.2.1. LF/HF Ratio

For each 5-min RR interval segment, the power spectral density was obtained using Burg’s algorithm, among which the components ranged from 0.04 Hz to 0.15 Hz and 0.15 Hz to 0.4 Hz and were extracted as the LF and HF power. The LF/HF ratio, the widely accepted feature to estimate the sympathetic/parasympathetic balance [27], is calculated as follows:

(1) $$\mathrm{LF}/\mathrm{HF}\;\mathrm{ratio}\;=\;\frac{\mathrm{LF}\;\mathrm{power}}{\mathrm{HF}\;\mathrm{power}}.$$

#### 2.2.2. TE

RR interval segments were filtered with Chebyshev Type II band-pass filter to separate LF components and HF components out, as time series indicating sympathetic and parasympathetic activity, respectively. Then, the TE in two directions, from LF to HF and from HF to LF, were calculated to quantify the directional information transfer between these two series.

Given time series $X\;=\;\left\{x_{1}{,\;x}_{2},\cdots {,\;x}_{N}\right\}$ and $Y\;=\;\left\{y_{1}{,\;y}_{2},\cdots {,\;y}_{N}\right\}$, we calculate transfer entropy from *X* to *Y* according to the following formula:

(2) $${\mathrm{TE}}_{X\to Y}\;=\;\sum_{x_{i}{,x}_{i-1}{,y}_{i-\tau }}p\left(x_{i}{,x}_{i-1}{,y}_{i-\tau }\;\right)\log\frac{p\left(x_{i}{,x}_{i-1}{,y}_{i-\tau }\;\right)p\left(x_{i-1}\right)}{p\left(x_{i-1}{,y}_{i-\tau }\;\right)p\left(x_{i}{,x}_{i-1}\right)},$$

where $p\left(x_{i},x_{i-1},y_{i-\tau }\;\right)$ is the joint probability for the occurrence of $x_{i}$, $x_{i-1}$ and $y_{i-\tau }$; i is the time subscript; and τ is the time lag. In this paper, we selected kernel density estimation to estimate the joint probability because it outperforms the fixed-binning method [28]. For instance, the joint probability $p\left(x_{i},x_{i-1},y_{i-\tau }\;\right)$ could be obtained through the following:

(3) $$p\left(x_{i},x_{i-1},y_{i-\tau }\right)\approx \frac{1}{P}\sum_{j=1}^{P}\frac{1}{h_{x_{i}}h_{x_{i-1}}h_{y_{i-\tau }}}K\left(\frac{x_{i}-x_{i,j}}{h_{x_{i}}}\right)K\left(\frac{x_{i-1}-x_{i-1,j}}{h_{x_{i-1}}}\right)K\left(\frac{y_{i-\tau }-y_{i-\tau ,j}}{h_{y_{i-\tau }}}\right),$$

where P is the number of data points; K is a “kernel function” and $h_{\left(.\right)}$ is the bandwidth of the kernels which are both described below; $y_{i-\tau ,j}$ indicates the j element of Y with a time lag of τ. Among a range of commonly used kernel functions, we chose Gaussian kernel:

(4) $$K\left(u\right)=\frac{1}{\sqrt{2\pi }}e^{-0.5u^{2}},$$

The bandwidth $h_{\left(.\right)}$, according to Silverman’s rule of thumb, was specified as:

(5) $$h_{\left(.\right)}=1.06{\alpha \hat{\sigma }P}^{-1/5},$$

where $\hat{\sigma }$ is an estimate of the standard deviation of the sample and *α* is a multiplier for scaling.

#### 2.2.3. Simulation

In all the above-mentioned formulas, time lag τ and multiplier α were undefined. 

To determine the value of α, a simulation was carried out. Simulated data *X* was designed as the driving system and *Y* as the responding system. These two sequences were oscillatory and are generated as follows [29]:

(6) $$x_{n}=1-\beta x_{n-1}^{2}+du_{n},\;y_{n}=\left(1-C_{1}\right)\left(1-\beta y_{n-1}^{2}\right)+C_{1}\left(1-\beta x_{n-1}^{2}\right)+du_{n},$$

where $u_{n}$ is a Gaussian noise; in order to mimic the time series LF component and HF component, the frequency of *X* and *Y* were specified to be 0.04~0.15 Hz and 0.15~0.4 Hz, respectively; *C*_1_ = 0.3 is the coupling coefficient and *d* = 0.03 is the coefficient of the noise amplitude, *β* = 1.8. Note, that in this equation, the time lag *τ* between *X* and *Y* was determined to be 1. 

One-hundred pairs of simulated consequences of *X* and *Y* were randomly generated according to Equation (6). Each consequence contained *n* = 512 points. For each pair of simulated data, we computed TE from *X* to *Y* (TE(*X*→*Y*)) and from *Y* to *X* (TE(*Y*→*X*)) with different value of α. The parameter α was varied from 0.5 to 7.0 in steps of 0.5. 

Then, to determine the value of τ, we calculated the TE between LF and HF of the RR interval segments of the normal group, with τ varied from 1 to 7 in steps of 1. In this part, we chose the optimal value of α obtained in the abovementioned simulation.

### 2.3. Performance Evaluation

For every computed feature, one-way analysis of variance (ANOVA) was implemented to verify whether any statistically significant difference existed via SPSS software. The features were considered to show significant differences between normal and CHF groups with a p value lower than 0.05. In this study, we carried out four discriminant analyses using SPSS. In the four analyses, there were two groups: normal group and CHF group. The input (independent indices) in the four analyses were: (1) LF/HF ratio for all subjects; (2) TE(LF→HF) for all subjects; (3) TE(HF→LF) for all subjects; (4) the combination of LF/HF ratio, TE(LF→HF) and TE(HF→LF) for all subjects.

## 3. Results

### 3.1. Parameter Selection

Among every pair of data, *X* is the driving system and *Y* is the target system. Since TE could quantify information exchanging among two systems [13], we supposed a smaller value of TE(*Y*→*X*) than that of TE(*X*→*Y*) in this simulation. To visualize to which degree TE(*Y*→*X*) was smaller than TE(*X*→*Y*), we proposed the ratio of TE(*Y*→*X*) to TE(*X*→*Y*) as an estimator of the performance in this simulation. A smaller ratio value (all ratios were smaller than 1) means that TE from the target system to driving system was much less than the opposite direction, thus indicating that the TE is sensitive to the disparity of the amount of information transfer in the two directions. In this case, TE presented a stronger ability to distinguish directed information flows. 

**Figure 2:**
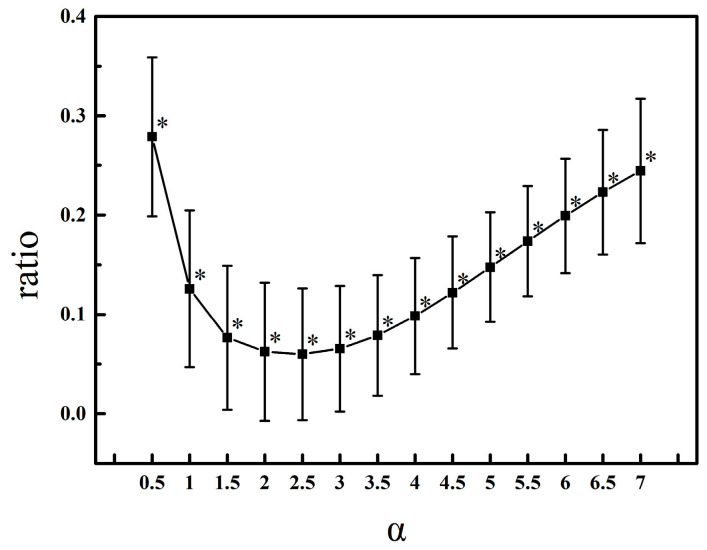
A plot of mean TE(*Y*→*X*)/TE(*X*→*Y*) ratio of 100 pairs of simulated consequences vs. multiplier *α*. * indicates highly significant difference between TE(*Y*→*X*) and TE(*X*→*Y*) with *p* < 0.001.

**Figure 3:**
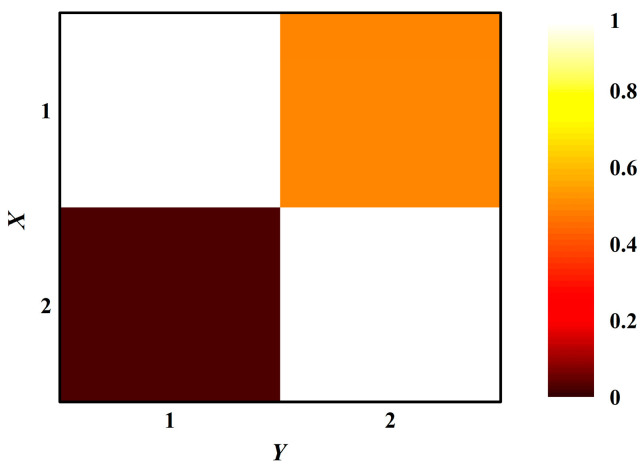
TE matrix representation for results of simulation. The color indicates the value of TE(1→2) corresponding to the colormap. The matrix shows mean values of TE(*X*→*Y*) and TE(*Y*→*X*) over 100 pairs of simulated data with *τ* = 1 and *α* = 2.5.

In this simulation, TE(*X*→*Y*) and TE(*Y*→*X*) were calculated for each pair of simulated data to choose a proper value of *α* with time lag *τ* set to 1 [20]. Figure 2 shows the performance of the simulation when *α* varies from 1 to 7. For each *α* value, extremely significant difference existed between TE(*X*→*Y*) and TE(*Y*→*X*), correctly distinguishing the driving and responding elements. When *α* = 2.5, the ratio of TE(*Y*→*X*) to TE(*X*→*Y*) reached its minimum, where TE shows the best discriminating ability. Figure 3 presents the mean value of TE in two directions in the color map to indicate the causality between processes *X* and *Y* when *τ* = 1 and *α* = 2.5. TE(*X*→*Y*) has a much higher average than TE(*Y*→*X*), indicating that these parameters are appropriate for TE to detect the direction of information transfer.

**Figure 4:**
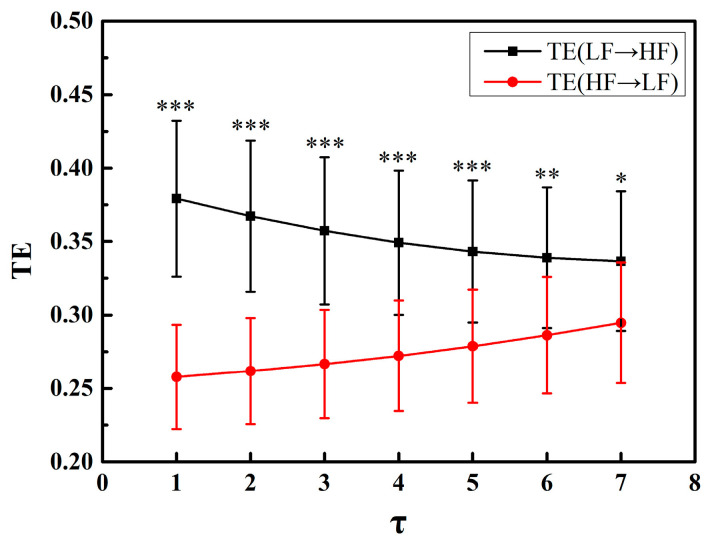
Plots of mean TE(LF→HF) and mean TE(HF→LF) vs. time lag *τ*. Significance of difference between TE(LF→HF) and TE(HF→LF) is presented as *, ** and ***, corresponding to *p* < 0.05, *p* < 0.01 and *p* < 0.001, respectively.

For all normal group RR interval segments, TE between LF and HF components was calculated and *α* was set to 2.5. Figure 4 shows the performances of TE(LF→HF) and TE(HF→LF) when *τ* varied from 1 to 7 in steps of 1. When *τ* = 1~5, TE in two directions showed an extremely significant difference, as *τ* increased, differences between TE(LF→HF) and TE(HF→LF) decreased and became less statistically significant, showing that the value of *τ* is gradually deviating from the true time lag between the LF and HF series. Among all values of *τ*, disparity between TE in two directions was largest when *τ* = 1, indicating that the time lag between the LF and HF series was closest to 1.

According to the results, we selected *τ* = 1 and *α* = 2.5, where TE exhibited the best discriminatory ability for directed information transfer.

### 3.2. Features Analysis

The LF/HF ratio was calculated to estimate the sympathetic/parasympathetic balance for the normal and CHF group. TE in two directions for normal and CHF groups were calculated with *τ* = 1 and *α* = 2.5. It is regarded as a powerful tool to explore the information exchanging among subsystems (the SNS and PNS).

**Figure 5:**
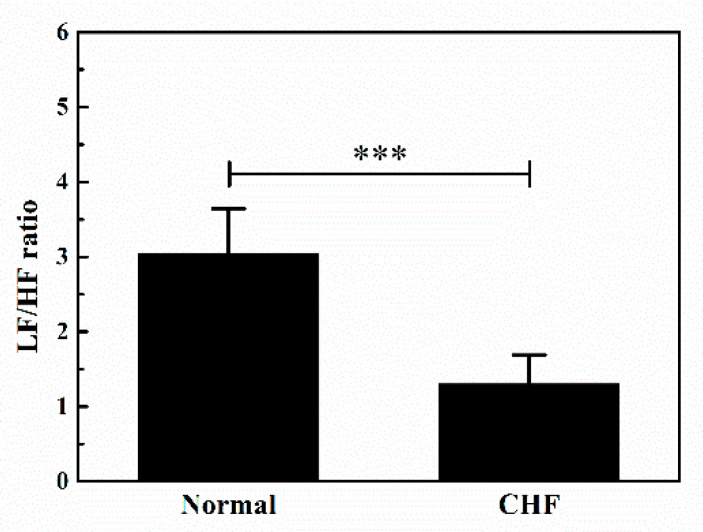
Mean and standard error of LF/HF ratio for the normal and CHF group. LF: power of low frequency component of RR intervals; HF: power of high frequency component of RR intervals. *** indicates *p* < 0.001.

Figure 5 presents the results of one-way ANOVA of the LF/HF ratio between the normal and CHF group. Compared with the normal group, the ratio of the CHF group is significantly lower. ANOVA confirmed an extreme statistical difference (*p* < 0.001) between the ratio of these two groups. The results reflect a sympathovagal imbalance in the CHF group, compared with the normal group.

**Figure 6:**
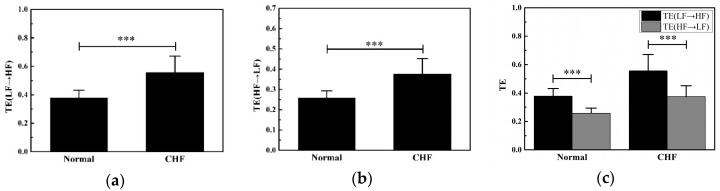
Performance of TE in the normal and CHF group. (**a**) Mean and standard error of TE(LF→HF) in two groups; (**b**) mean and standard error of TE(HF→LF) in two groups; (**c**) mean and standard error of TE in two directions in two groups. TE(LF→HF): transfer entropy from the low frequency component to high frequency component of the RR interval segment; TE(HF→LF): transfer entropy from high frequency component to low frequency component. *** indicates *p* < 0.001.

Results of TE are shown in Figure 6. In Figure 6a,b, the CHF group has higher value of both TE(LF→HF) and TE(HF→LF) than the normal group. For two directions, an extremely significant difference (*p* < 0.001) exists in TE among two groups as ANOVA showed. In Figure 6c, averages of TE(LF→HF) and TE(HF→LF) of normal and CHF groups are presented. For each group, TE(LF→HF) has a higher value than that TE(HF→LF). Statistically, there is an extremely significant difference (*p* < 0.001) existing between the two directions of TE in each group.

### 3.3. Classification

Samples used during CHF recording screening were obtained by calculating the mean value of all the 5 min segments for each recording. Fisher’s discriminant function (applied with SPSS software) classified each sample as normal or CHF. Classification based on LF/HF ratio and TE in two directions was carried out by linear discriminant analysis. Accuracy, sensitivity and specificity achieved by the classification, adopting different features, are demonstrated in Table 2. 

**Figure 7:**
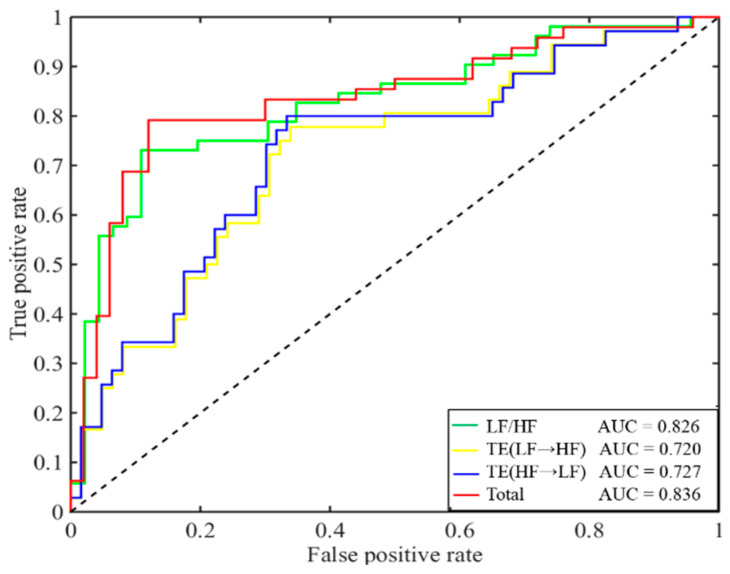
ROC curves for the LF/HF ratio, TE(LF→HF), TE(HF→LF) and a combination of the three features.

Based on the combination of the three features (LF/HF ratio, TE(LF→HF) and TE(HF→LF)), the classification reached its best performance, compared with discriminating based on features separately. The performances of CHF’s classifications were described by receiver operating characteristic (ROC) curves, as shown in Figure 7. The combination of the three features led to the best performance, where the area under the curve (AUC) was 0.836.

## 4. Discussion

### 4.1. Comparison and Summary

Heart rate variability (HRV) depicts the functional status of the autonomic nervous system and has been introduced in patients with congestive heart failure (CHF). Galinier et al. reported that HRV variables such as SDNN and SDANN were significantly lower in CHF patients than in controls, reflecting the depression of HRV in CHF subjects [30]. Lopera et al. found that the HRV analyzed in the time and frequency domain reduced in patients with CHF [31]. İşler et al. reported that the combination of the classical HRV indices with indices obtained from wavelet entropy calculations could significantly improve the performance of the HRV analysis in CHF screening [32]. In this paper, the LF/HF ratio is significantly distinguished between the normal group and CHF group (Figure 5), which is consistent with other findings studying HRV features of CHF [33,34,35,36]. However, they only focused on decomposing independent frequency components of HRV but neglected a complex interaction between the independent frequency components of HRV. 

Considering that TE can quantify the amount of nonlinear interaction between two systems, in this study, it was used for interaction analysis in two subsystems of ANS. Both directions between SNS and PNS showed a significant difference between the normal group and CHF group (Figure 6). 

**Figure 8:**
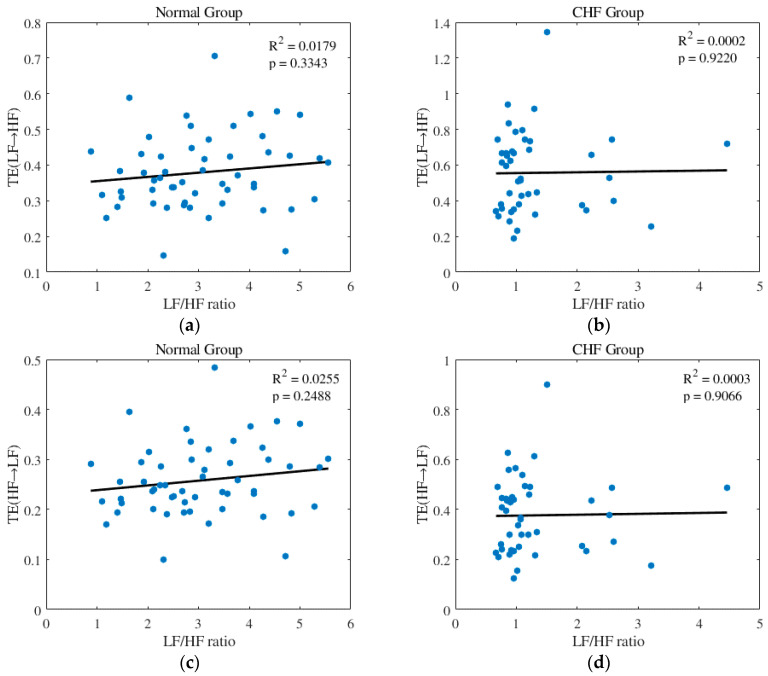
Results of correlation analysis: (**a**) Scatterplot of TE(LF→HF) with the LF/HF ratio in the normal group; (**b**) Scatterplot of TE(LF→HF) with the LF/HF ratio in the CHF group; (**c**) Scatterplot of TE(HF→LF) with the LF/HF ratio in the normal group; (**d**) Scatterplot of TE(HF→LF) with the LF/HF ratio in the CHF group.

We carried out the correlation analysis between TE(LF→HF)/TE(HF→LF) and the LF/HF ratio. However, as the results show in Figure 8 below, in both CHF and normal groups, no significant correlation between TE(LF→HF) and the LF/HF ratio is shown, nor between TE(HF→LF) and the LF/HF ratio. We assumed that it was because TE was time-related, as it calculates the relationship between the two time series for each time point, while the LF/HF ratio is not. Plus, TE helps improve the screening accuracy of the LF/HF ratio from 79.6 to 83.7%. Thus, we think that TE is capable of providing more information in addition to the LF/HF ratio.

### 4.2. Physiological Significance

It has been reported that CHF is a chronic cardiovascular syndrome which is characterized by an imbalance of activity in the autonomic nervous system [24,37,38]. The HRV analysis is a widely used method to investigate the ANS modulation of the heart [20,39,40]. To investigate the balance of ANS activity, the LF/HF ratio is a more robust value in HRV frequency domain analysis. The LF/HF ratio showed a significantly lower value in the CHF group compared with the normal group, revealing that the equilibrium of the ANS in CHF patients was disturbed. Considering that the TE method can convey directional information between two biomedical time series, TE was further adopted to study the information interaction between two independent subsystems of ANS [14].

Transfer entropy can quantify the amount of nonlinear interactions between two systems, it estimates the amount of uncertainty reduced in future values of one time series by knowing the past values of another series [41]. It has been shown that the two divisions of the ANS have antagonistic effects on the performance of the heart, while these opposing influences are based on complicated interactions existing in ANS [20]. In this paper, the TE(LF→HF) and TE(HF→LF) are both significantly higher in the CHF group than in the normal group (Figure 6), revealing a larger amount of information exchanging between LF and HF components existing in CHF patients. As LF and HF components could reflect the SNS and PNS activity respectively, it could be inferred that the interaction between SNS and PNS in CHF patients is strengthened. It is known that the biological systems sought to maintain a constant output after perturbation [42]. Considering that the LF/HF ratio reduced in the CHF group, the stronger interaction between SNS and PNS in CHF patients may be derived from the increased instability of ANS. In addition, insufficient blood flow to the heart of CHF patients may result in increased interaction between autonomic nerves to preserve their autonomic function [43,44]. Moreover, the TE(LF→HF) in the CHF group is significantly higher than TE(HF→LF) in the CHF group, which may indicate that the information transmission from SNS to PNS plays a major role. Thus, as a noninvasive non-linear indicator, it provides a new insight into the interval complicated interaction of autonomic nerve activities.

### 4.3. Parameter Discussion

The time lag *τ* at which significant coupling exists, is not known a priori. In this paper, the time lag τ was set to 1 and multiplier α was set to 2.5. In the simulation, the average TE of *Y*→*X* is much higher than the average TE of *X*→*Y* (Figure 3). The simulation indicated that it was reasonable to set τ and α at 1 and 2.5 in this study for correct information transfer, which is consistent with previous studies [28]. When *α* varied from 1 to 7 in steps of 0.5 in the simulated experiment, results revealed that the mean TE(*Y*→*X*)/TE(*X*→*Y*) ratio value reached its minimum with the *α* setting at 2.5 (Figure 2). A smaller ratio value means that TE is more sensitive to the disparity of the amount of information transfer in the two directions, where TE presented a stronger ability to distinguish directed information flows. Considering that the parameter selection may change much more with real-life data, the TEs between the LF and HF components were calculated for all RR interval segments of the normal group with *τ* varying from 1–7. Results showed that the TE in two directions showed an extremely significant difference with *τ* = 1, α = 2.5 (*p* < 0.001, Figure 6). Thus, it was suitable for *τ* and *α* to be set to 1 and 2.5 in this CHF data.

This study had some limitations. First, the ages of these subjects are not uniform, though not significantly different, which may have some influence on the results. Second, whether or not the subjects showed atrial fibrillation was unknown since differences caused by various complications of CHF were not our main concern in this study. Further exploration on the effects of different complications on ANS activity may be discussed in our further study. Third, although the LF component is widely accepted as a combination of PNS and SNS activity, we used the LF component to reflect SNS activity because SNS activity accounts for the dominance of the LF component as some studies reported in order to simplify the process of interaction between SNS and PNS. Additionally, although the LF/HF ratio and TE can significantly differentiate between the normal and CHF group, the severity of heart failure was not considered. Finally, respiratory effects on heart rate variability were not included in our estimation. Therefore, we will consider these limitations in the future.

## 5. Conclusions

This paper explored the interaction between the SNS and PNS in CHF by using the TE method. The results indicated that TE is a useful maker to detect ANS modulation between SNS and PNS. Moreover, the interaction of the two subsystems is strengthened in CHF patients compared with normal people, and the SNS is dominant in the information transmission of ANS. Therefore, the TE provides a novel insight into the interaction analysis of the ANS.

## Figures and Tables

**Table 1:** Details about the used data obtained from PhysioNet.

Database	NSR (*n* = 54)	BIDMC (*n* = 15)	CHF (*n* = 29)
Gender (M/F/U)	31/23	11/4	8/2/19
Age (years)	61.38 ± 11.63	55.51 ± 11.44	
NYHA-Class: *n*	Normal: 54	I, II, III: 4, 8, 17	III~IV: 15

NSR: Normal Sinus Rhythm RR Interval Database; BIDMC: BIDMC Congestive Heart Failure Database. CHF: congestive heart failure.

**Table 2:** Performance of classification.

Indices	Acc (%)	Sen (%)	Spe (%)
LF/HF ratio	79.6	86.4	74.1
TE(LF→HF)	69.4	56.8	79.6
TE(HF→LF)	70.4	56.8	81.5
All features	83.7	86.4	81.5

Acc: accuracy; Sen: sensitivity; Spe: specificity. LF/HF ratio: ratio of power of the low frequency component to the high frequency component of RR intervals; TE(LF→HF): transfer entropy from the low frequency component to the high frequency component of the RR interval segment; TE(HF→LF): transfer entropy from the high frequency component to the low frequency component. All features: linear discriminant analysis based on both LF/HF ratio, TE(LF→HF) and TE(HF→LF).
